# Data on the optimisation of a solid phase extraction method for fractionating estrogen metabolites from small urine volumes

**DOI:** 10.1016/j.dib.2020.105222

**Published:** 2020-02-03

**Authors:** Carien L. van der Berg, Gerda Venter, Francois H. van der Westhuizen, Elardus Erasmus

**Affiliations:** Human Metabolomics, North-West University (Potchefstroom Campus), Potchefstroom, 2531, South Africa

**Keywords:** Estrogen metabolism, Solid phase extraction, LC-MS/MS, Method optimisation, LC, Liquid chromatography, MS, Mass spectrometry, MS/MS, Tandem mass spectrometry, OH, Hydroxy, M, Methyl conjugation, S, Sulphate conjugation, G, Glucuronide conjugation, MeOH, Methanol, v/v, Volume/volume ratio, E1, Estrone, E2, Estradiol, E3, Estriol, ESI, Electrospray ionisation, SPE, Solid phase extraction

## Abstract

Certain estrogen metabolites have been implicated in the pathophysiology of breast cancer. Moreover, the estrogen metabolite profiles of healthy women and those with (a high risk of) breast cancer differ significantly. The development of an analytical method to determine the relative levels of all the estrogen biotransformation products has been described in van der Berg et al. [1]. An improvement on previously developed methods was the ability to also detect molecules such as sulphate and glucuronide conjugates as well as progesterone, estradiol precursors, and metabolites from the 16-hydroxylation metabolic pathway of estrogens simultaneously with all other estrogen metabolites. The data presented here describe the optimisation of a solid phase extraction method with different fractionation steps for LC-MS/MS analysis of 27 estrogen-related metabolites from small urine volumes. Conditions that were optimised include the elution and washing solvent concentration, the urine, loading, washing, and elution volumes, as well as pH. All raw data used to construct the bar graphs presented in this article are included in the supplementary data file. The data indicated that fractionation was necessary in order to elute estrogen metabolites with different chemical properties at different eluate compositions. Only one of the fractions (containing the less water-soluble metabolites) underwent derivatisation before LC-MS/MS analysis.

**Table:** Specifications Table

Subject	Biochemistry
Specific subject area	Metabolite extraction for estrogen biotransformation analysis
Type of data	Table Graph
How data were acquired	Liquid chromatography mass spectrometry using an Agilent 1290 Infinity series LC system (Agilent Technologies, Santa Clara, CA, USA) coupled to the Agilent 6460 triple quadrupole mass spectrometer (G6460A)
Data format	Raw Analysed
Parameters for data collection	A number of parameters for solid phase extraction (SPE) were optimised, including the elution and washing solvent concentration, the urine, loading, washing, and elution volumes, as well as pH.
Description of data collection	Optimisation of the sample preparation method was done by varying a certain SPE parameter while keeping all other parameters unchanged and analysing chromatographic responses of the metabolites in the eluant.
Data source location	Potchefstroom, South Africa, 26°41′16.5″S 27°05′33.0″E
Data accessibility	With the article
Related research article	C. Van der Berg, G. Venter, F.H. Van Der Westhuizen, E. Erasmus, Development and Validation of LC-ESI-MS/MS Methods for Quantification of 27 Free and Conjugated Estrogen-Related Metabolites, Analytical Biochemistry, DOI: 10.1016/j.ab.2019.113531

**Table:** 

**Value of the Data** • The data could be used by other researchers to help with overall SPE method development since the effect of certain parameters is illustrated. • The data could be useful for the development or optimisation of steroid metabolite- specific SPE methods in different labs that use different LC-MS/MS instruments for detection. • The data indicate that LC-MS/MS analysis of low concentration metabolites might be improved by fractionation of the eluates.

## 1 Data description

The data presented here were obtained during the development of an LC-ESI-MS/MS method for the detection and quantification of various urinary estrogen metabolites [1]. A number of conditions for solid phase extraction (SPE) were tested by determining the response of estrogen-related metabolites on the LC-MS/MS after each adjustment. Before LC-MS/MS analysis, SPE eluates were dried and dansyl derivatised (if applicable). The integrated chromatographic spectral results are available as raw data tables in the supplementary file accompanying this article. This data was plotted as response or log transformed response values over different variables. The composition of the estrogen metabolite mixture that was used for these assays is given in Table 1 in the Materials and Methods section. Due to the large variation in the water solubility of estrogen metabolites and the fact that the sulphate and glucuronide conjugates (most water soluble) do not dansyl derivatise, we considered the possibility of analysing the derivatising and non-derivatising metabolites separately (i.e. by fractionating the sample).

**Fig. 1:**
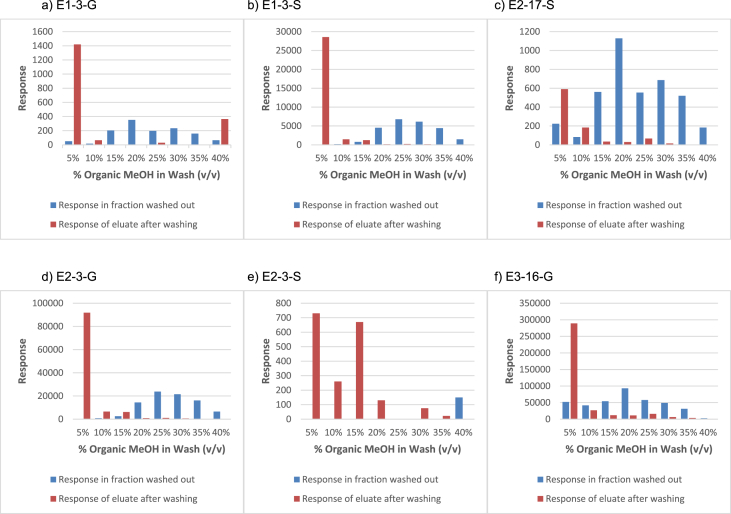

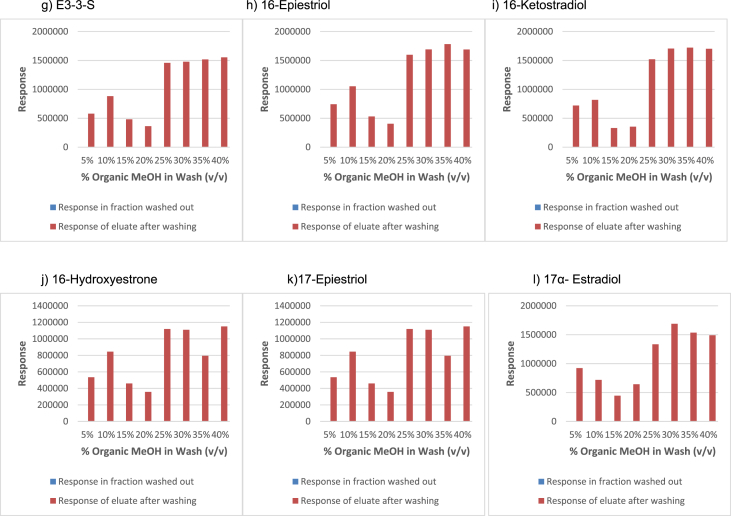

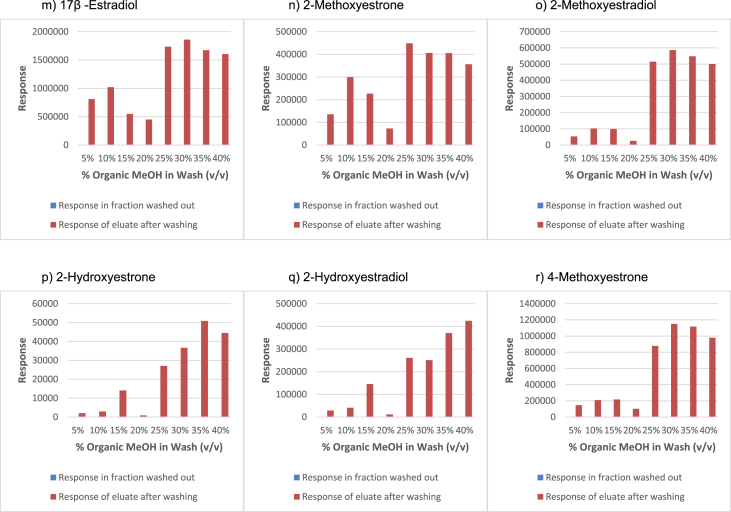

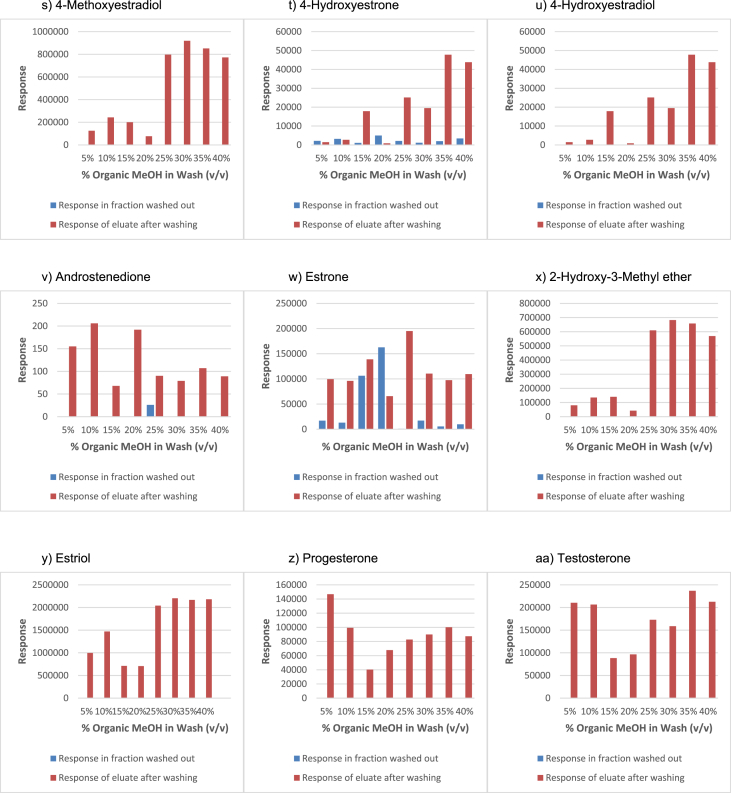
**(a–aa): Relative responses of the individual steroid hormone metabolites on the mass spectrometer of both the wash and elution steps after washing with different concentrations of methanol.** Washing was done with 5%, 10%, 15%, 20%, 30%, 35%, and 40% (v/v) methanol (MeOH) and the washed-out fractions were analysed. This was followed by an elution step with methanol and acetone, followed by the analysis of the eluate. Blue bars represent the response of metabolites washed from the SPE column, while the red bars represent the response of the metabolites that eluted with methanol and acetone at the end of the sample preparation method.

**Fig. 2:**
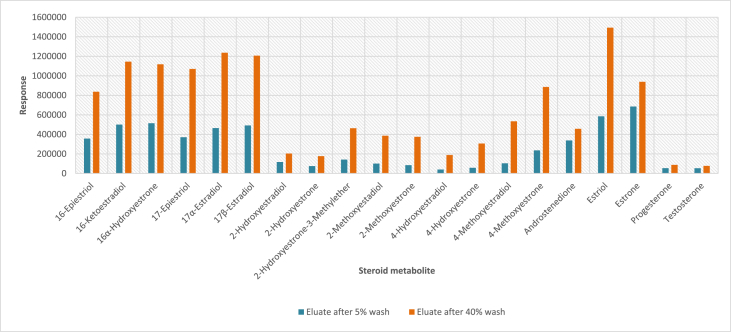
**Relative responses of each of the final eluate metabolites on the mass spectrometer after washing only with 5% MeOH or first with 5% and then 40% (v/v) MeOH.** Blue bars represent the response of the eluate after a single 5% MeOH in water wash, followed by elution with methanol and acetone (i.e. no fractionation, conjugates co-eluted with other metabolites). Orange bars represent the response of the SPE eluates after first washing with 5% MeOH, eluting conjugates with 20% MeOH, washing a second time with 40% MeOH, and finally eluting the remaining metabolites with methanol and acetone.

**Fig. 3:**
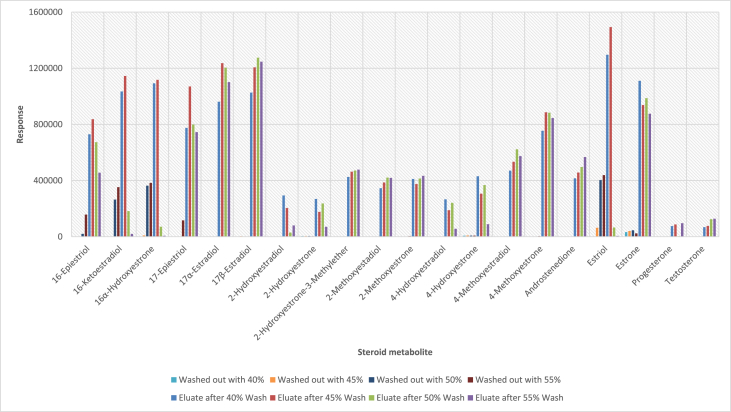
**Relative responses on the mass spectrometer of the SPE eluate after a second wash with 40%, 45%, 50% and 55% (v/v) MeOH in the SPE washing solution for each of the less water-soluble steroid hormone metabolites.** The first four bars represent the response in the washing solution whereas the last four bars for each metabolite indicate the response in elution fractions.

**Fig. 4:**
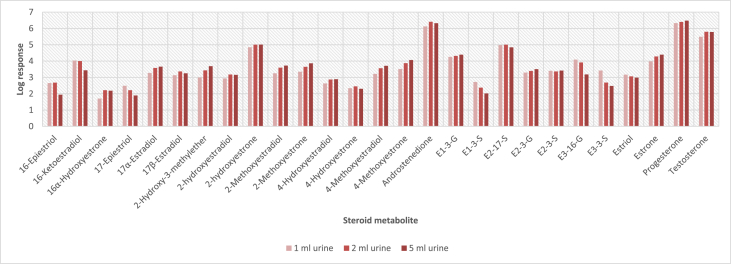
**Log transformed mass spectrometric responses for urine volumes of 1 ml, 2 ml, and 5 ml for each steroid hormone metabolite.** These urine samples were spiked and cleaned through C18 SPE before being analysed on LC-MS/MS.

**Fig. 5:**
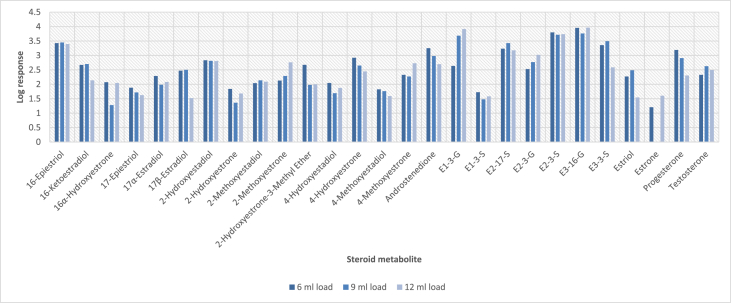
**Log transformed LC-MS/MS response for each steroid metabolite with different loading volumes.** Loading volumes of 6 ml, 9 ml and 12 ml, consisting of 1 ml samples diluted with 5 ml, 8 ml or 11 ml water respectively, were compared.

**Fig. 6:**
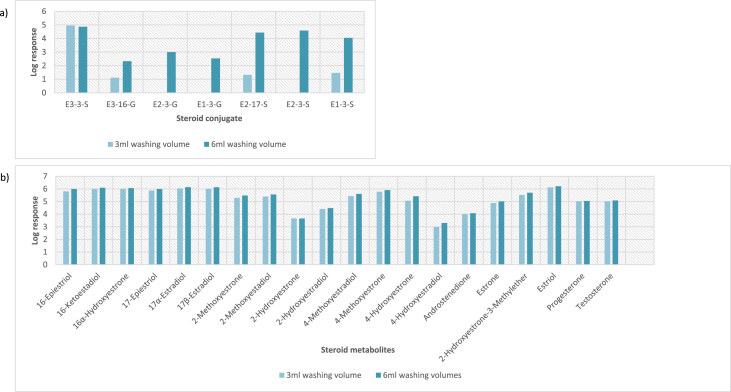
**Comparison of the effect of washing with 3 ml or 6 ml washing volumes for the a) underivatised conjugated molecules and b) dansyl derivatised second SPE eluate.** The log values of the responses were plotted for each steroid metabolite. These washing steps include the first 5% (v/v) wash, first 20% (v/v) elution step (highly water-soluble conjugates), and the second 45% (v/v) wash. These relative responses of each metabolite indicate an effect of the washing volumes, especially for sulphate and glucuronide conjugates.

**Fig. 7:**
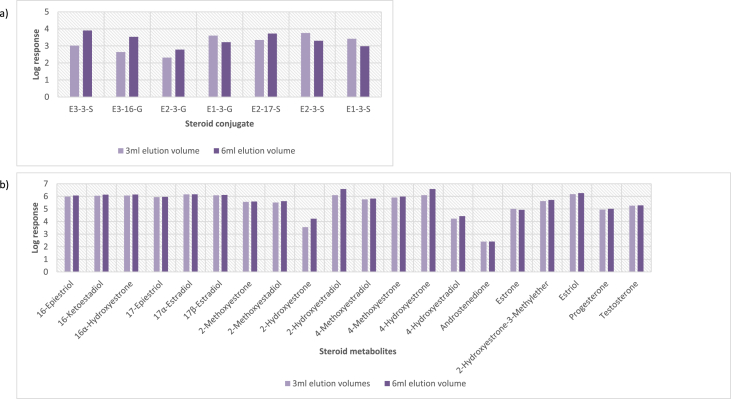
**Comparison of the effect of eluting with 3 ml or 6 ml for the two different elution steps for the a) underivatised conjugated molecules and b) dansyl derivatised second SPE eluate.** The log values of the responses were plotted for each steroid metabolite. These elution volumes included a 2:4 ratio of methanol:acetone. The relative responses of each metabolite indicate an effect of the elution volumes, especially on the hydroxyestrogens.

**Fig. 8:**
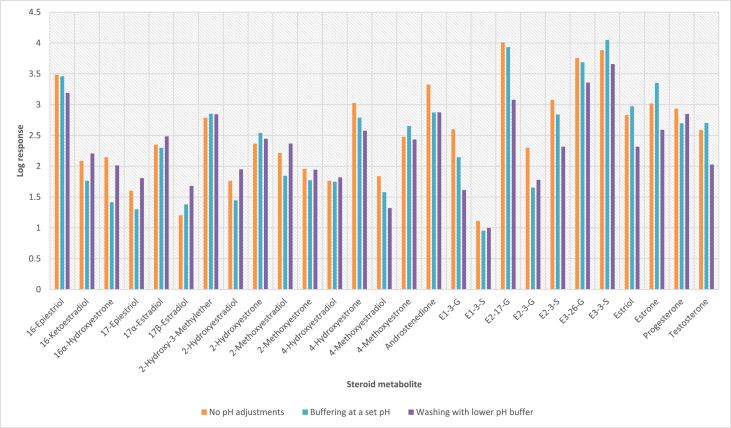
**Log transformed response values of the steroid hormone metabolites to illustrate the effect of varying pH levels on the responses for each of the 27 estrogen metabolites.** No pH adjustments (normal water and methanol) are indicated in orange, adjusting the pH to 7 and using a buffer (ammonium formate) is indicated in blue, whereas purple bars indicate a more acetic wash and alkaline elution.

**Fig. 9:**
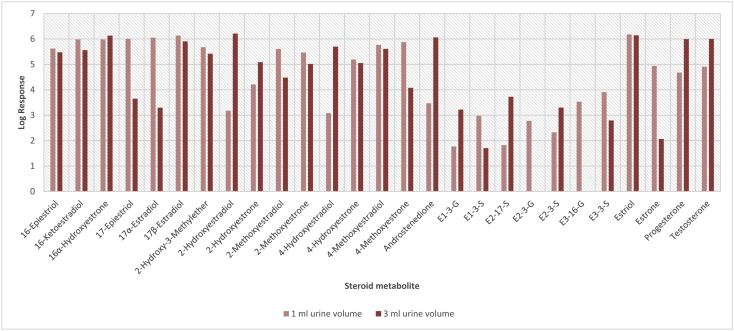
**Log transformed mass spectrometric responses for urine volumes of 1 ml and 3 ml after all other washing and elution volumes were optimised.** These urine samples were spiked and cleaned through C18 SPE before being evaluated on LC-MS/MS.

In order to determine the optimal conditions to fractionate the samples before derivatisation, we first tested different washing and elution solvent concentrations. The plotted bar graphs of the response of each metabolite at different (5–40%) organic solvent concentrations in the washing solution are presented in Fig. 1 a-aa. The raw data used to compile the graphs shown in Fig. 1 are available in Table 1 of the supplementary data file. Fig. 2 shows the response after a second 40% methanol wash, while Fig. 3 shows a comparison of the responses after washing with different methanol compositions (40–55%). The raw data of Fig. 2, Fig. 3 are available in Supplementary Table 2. In addition, the urine and SPE loading, washing, and elution volumes, as well as the pH, were optimised. The log transformed data presented in Fig. 4 (raw data in Supplementary Table 3) show the metabolite responses when 1 ml, 2 ml or 5 ml urine was used. Fig. 5 shows the effect of different SPE loading volumes on the metabolite responses while the raw data before log transformation is available in Supplementary Table 4. The log transformed responses of the conjugated (Fig. 6a) and the unconjugated (Fig. 6b) metabolites after washing with either 3 ml or 6 ml was also determined. The graphs were plotted separately because the metabolites in Fig. 6a underwent only a single 5% washing step, whereas the metabolites in Fig. 6b underwent multiple washing steps (5%, and 45%). Furthermore, metabolite responses were compared using different elution volumes (3 ml or 6 ml; Fig. 7). The raw data supporting Figs. 6 and 7 is given in Supplementary Table 5. Finally, in Fig. 8 the effect of the pH of the buffer or washing and elution steps on the response values can be seen. Fig. 8 represents the log values of the raw data found in Supplementary Table 4. Another urine volume comparison was done (Fig. 9) to confirm that, after all other parameters were optimised, the most optimal urine volume was chosen (raw data in Supplementary Table 6).

## 2 Experimental design, materials, and methods

Hormone analytical standards were purchased from Sigma Aldrich (St. Louis, MO): estrone (E1), 17α-estradiol (E2), 17β-E2, estriol (E3), progesterone, testosterone, androstenedione, E3-3-sulphate (S), E2-3-S, E1-3-S, E1-3-glucuronide (G), E2-3-G, E3-16-G. Analytical standards for 2-hydroxy(OH)-E1, 4-OHE1, 16α–OH–E1, 2-methoxy(M)E1, 4-ME1, 2-OHE2, 4-OHE2, 2OHE1-3-methyl, 2-ME2, 4-ME2, 16-epiestriol, 17-epiestriol, 16-ketoE2, and E2-17-S and internal standards in the form of isotopes of E1, E2, E3 and progesterone were purchased from Steraloids (Newport, RI). Isotopes of 2- and 4-OHE1 to be used as internal standards were purchased from Toronto Research Chemicals (North York, Canada). General use compounds – dansyl chloride, ammonium formate and 2-acetamidophenol, as internal standard – were purchased from Sigma Aldrich. Lastly, all the high-purity Burdick and Jackson Brand® solvents and water used on the liquid chromatography (LC)–tandem mass spectrometry (MS/MS) instrument were obtained from Honeywell Research Chemicals (Bucharest, Romania). For the sample preparation, a Phenomenex (Torrance, CA) SPE column vacuum manifold (AHO-6024) with SPE cartridges Phenomenex Strata C18-E (8B–S002-FBJ) was used.

### 2.1 Working solution preparations

The stock solutions of all the estrogens (Table 1) were prepared at concentrations of 0.1 mg/ml in methanol, and further diluted to 50 ng/ml working stock solutions in MeOH. The standards were then stored at −80 °C until used for method development. For spiked urine solutions, the same concentration was spiked in a single urine sample to be used for all analysis.

**Table 1:** All the steroid hormone metabolites included in the stock solution used for SPE optimisation.

16-Epiestriol	Estradiol 17-sulphate	2-Hydroxyestradiol
16-Ketoestradiol	Estradiol-3-glucuronide	2-Hydroxyestrone
16α-Hydroxyestrone	Estradiol-3-sulphate	2-Hydroxyestrone-3-methyl ether
17-Epiestrol	Estriol-16 glucuronide	2-Methoxyestradiol
17α-Estradiol	Estriol-3-sulphate	2-Methoxyestrone
17β-Estradiol	Estrone-3-glucuronide	4-Hydroxyestradiol
Estriol	Estrone-3-sulphate	4-Hydroxyestrone
Estrone	Testosterone	4-Methoxyestradiol
Androstenedione	Progesterone	4-Methoxyestrone

### 2.2 LC-MS/MS analysis

Liquid chromatography mass spectrometry was performed using an Agilent 1290 Infinity series LC system (Agilent Technologies, Santa Clara, CA, USA) consisting of a micro vacuum degasser (G1330B); binary pump (G4220A); preparative autosampler HiP-ALS (G4226A); and thermostatted column compartment (G1216C). The LC system was coupled to the Agilent 6460 triple quadrupole mass spectrometer (G6460A), with a jet stream electrospray ionisation (ESI) source. The Agilent Technologies, Zorbax Eclipse plus rapid resolution high definition (RRHD) C8-E chromatographic column (959758–906), were used for liquid chromatographic separations. The LC-MS/MS methods as described in Van der Berg et al. (2020) was followed for sample analysis [1]. These methods consisted of two different runs, one for the more water soluble and one for the less water soluble metabolites.

### 2.3 Solid phase extraction method outline

The general solid phase extraction (SPE) sample preparation method was followed. The adsorbent in the SPE cartridges (Strata C18-E) was conditioned and equilibrated with two cartridge volumes of each, acetone, methanol (MeOH), and water at a flow rate of 3 ml/min. The adsorbent bed was not allowed to run dry before the next step, which included sample loading. Before loading, the samples (standard or matrix) were centrifuged and only the supernatant was diluted with water to a total volume of twice the SPE column capacity, and, unless indicated otherwise, the pH was adjusted to 7. The sample was then loaded onto the adsorbent. The adsorbent was washed with single or multiple water or higher organic composition solvents and the adsorbent was allowed to dry for 10 min under high vacuum. The last step of the SPE included the elution of the metabolites of interest into a polypropylene tube at approximately 1 ml/min with at least two column volumes of methanol or acetone. Any of the collected washing or eluate fractions containing solvent were dried under a gentle stream of nitrogen gas and those that contained water were freeze-dried. If both were present, the organic solvent was evaporated first, followed by freeze-drying the remaining aqueous solution. The less water-soluble fractions were dansyl derivatised and the more water-soluble, conjugated fraction was resuspended in a 50:50 water:MeOH mixture before both fractions were filtered through 0.2 μm Nylon Spin-X filters for LC, after which the filtrate was transferred to inserts in LC-MS analysis vails.

### 2.4 Solid phase extraction method optimisation

#### 2.4.1 Washing and elution solvent

SPE optimisation was done using either a 50 ng/ml standard mixture prepared in methanol or a urine sample spiked with the same concentration (50 ng/ml) of estrogen metabolite mixture. To test the possible advantage of fractionation of the sample, standard estrogen metabolite mixtures were loaded onto SPE cartridges, which were then washed with 5%, 10%, 15%, 20%, 25%, 30%, 35% and 40% (v/v) organic solvent (MeOH) in the aqueous washing step to give an indication of which metabolites will elute at which stage. Both the wash and elution steps were collected, dried, derivatised (if applicable), and analysed using the LC-MS/MS method described in van der Berg et al. [1]. For confirmation if fractionating the samples in different elution steps (one for the more and a second for the less water-soluble metabolites) would increase responses, the following procedure was followed. The estrogen standard mixture spiked in urine was loaded on SPE columns, after which the first column was washed with 5% MeOH and eluted, and the second was washed with 5% MeOH followed by a first elution with 20% MeOH and a 40% MeOH second wash. To optimise the organic composition of the second washing step, standard estrogen metabolite mixtures were again loaded to the SPE columns and, first, washed with 5% (v/v) MeOH in water, followed by the first elution with 20% (v/v) methanol, and then a second wash with 40%, 45%, 50% or 55% (v/v) MeOH in water. Again, all fractions were collected and the response of the last eluting metabolites (unconjugated and dansylated metabolites) was plotted for comparison.

#### 2.4.2 Urine, loading, washing, and elution volumes

Following optimisation of the SPE washing and elution solvent composition, different urine, loading, washing and elution volumes were tested. For this, a urine sample spiked with 50 ng/ml standard mixture was loaded to SPE columns, washed with 5% (v/v) MeOH, then 20% (v/v) MeOH (elution 1), then 45% (v/v) MeOH, and metabolites finally eluted with methanol and acetone. The urine volumes that were tested included 1 ml, 2 ml and 5 ml. For the loading volume analysis, 1 ml urine was diluted to a final volume of 6 ml, 9 ml or 12 ml, while washing and elution volumes of either 3 ml or 6 ml water and methanol (premixed) were tested for all the washing and elution steps. The SPE eluates were dried and derivatised (if applicable) after sample clean-up and analysed in order to compare metabolite responses.

#### 2.4.3 pH

The use of a buffering agent, such as 10 mM ammonium formate, and different pH levels during different steps in the SPE procedure was also investigated. We compared (1) no pH buffering (in washing and elution steps), with (2) pH buffering at pH 7 with 10mM ammonium formate, and (3) conditioning with a more acetic solvent and subsequent elution with an alkaline solvent [2,3].

#### 2.4.4 Confirmation of optimal urine volume

Finally, the optimal urine volume for SPE was confirmed by applying the optimised conditions for all other SPE parameters. For this, the spiked urine samples were diluted to a 6 ml loading volume either by adding 5 ml of water to 1 ml of the urine sample, or by adding 3 ml of water to 3 ml of the urine sample. The SPE columns were conditioned with 6 ml acetone, 6 ml methanol and 6 ml distilled water. The prepared urine sample supernatant was then loaded onto the SPE columns. Each column was washed with 6 ml 5% (v/v) MeOH in water followed by a 6 ml 20% (v/v) MeOH in water, from which the sulphate and glucuronide conjugates were collected. The SPE cartridge was then washed again with a 45% (v/v) MeOH in water solution and dried under vacuum. The sample was next eluted with 2 ml MeOH and 4 ml acetone. The SPE elutes were then evaporated to dryness, resuspended or derivatised, analysed by LC-MS/MS, and the response of each metabolite was log transformed and plotted for comparison of the different urine volumes.
