# Supplementary material for: Data on the optimisation of a solid phase extraction method for fractionating estrogen metabolites from small urine volumes
Source: Data Brief. 2020 Feb 3;29:105222. doi: 10.1016/j.dib.2020.105222 (PMC7016251; doi:10.1016/j.dib.2020.105222)
Supplement: Multimedia component 1 [file mmc1.pdf]

Supplementary data for:

Data on the Optimisation of a Solid Phase Extraction Method for Fractionating Estrogen Metabolites from Small Urine Volumes

Authors: Carien L. van der Berg[a*], Gerda Venter[a], Francois H. van der Westhuizen[a], Elardus Erasmus[a]

Affiliations: [a] Human Metabolomics, North-West University (Potchefstroom Campus), Potchefstroom 2531, South Africa.

Contact email: *Corresponding author at Human Metabolomics, North-West University (Potchefstroom Campus), Potchefstroom 2531, South Africa. Carien.Jacobs@nwu.ac.za

This supplementary file contains the raw response values integrated for each metabolite that were used to produce Figures 1-9 in the data article: Data on the Optimisation of a Solid Phase Extraction Method for Fractionating Estrogen Metabolites from Small Urine Volumes. The abbreviation SPE used throughout the supplementary file refers to 'solid phase extraction'.

Supplementary Table 1. Raw response values obtained from LC-MS/MS chromatogram integration for different SPE washing conditions. Transition refers to the MS1-> MS2 m/z used to detect and give response values. These raw data were used for compiling Figure 1 in the data article.

Compound Name	Transition	Elution after 5% Wash Response	Elution after 10% Wash Response	Elution after 15% Wash Response	Elution after 20% Wash Response	Elution after 25% Wash Response	Elution after 30% Wash Response	Elution after 35% Wash Response	Elution after 40% Wash Response
16-Epiestriol	522.0 -> 171.0	579898	882309	481400	362891	1459100	1477674	1518819	1553509
16-Ketoestradiol	520.4 -> 171.1	742122	1052643	530946	405224	1598877	1691467	1783481	1689480
16α-Hydroxyestrone	520.4 -> 171.1	720758	818434	330722	355016	1520146	1705926	1721293	1702517
17-Epiestriol	522.0 -> 171.0	534326	845093	459478	357197	1118970	1110192	794227	1150568
17α-Estradiol	506.2 -> 171.1	922111	719090	445392	642837	1333363	1689277	1535275	1489710
17β-Estradiol	506.2 -> 171.1	811239	1021532	549652	450996	1735306	1860183	1672444	1604490
2-Hydroxy-3-Methylether	534.4 -> 171.1	135294	299622	226481	73141	448662	406128	405369	356414
2-Hydroxyestradiol	755.0 -> 170.9	2034	2929	14075	890	27062	36629	50853	44499
2-Hydroxyestrone	753.4 -> 170.1	28499	40846	145186	11359	260994	250764	370413	424480
2-Methoxyestradiol	536.4 -> 171.1	53400	102247	99125	25633	514939	586088	548181	500025
2-Methoxyestrone	534.4 -> 171.1	146247	208349	216967	101232	878074	1150621	1116708	979237
4-Hydroxyestradiol	755.0 -> 170.9	1453	2702	17868	851	25160	19488	47779	43833
4-Hydroxyestrone	753.4 -> 170.1	224868	381307	1961745	105699	3397430	2365351	5020764	5359140
4-Methoxyestradiol	536.4 -> 171.1	125073	243049	200098	76230	797146	919008	852146	772610
4-Methoxyestrone	534.4 -> 171.1	80187	135204	140236	42218	609924	682725	658226	569132
Androstenedione	287.2 -> 97.1	155	206	68	192	90	79	107	89
Estradiol-17-sulphate	351.2 -> 97.0	8169	604	461	53	101	43	0	0
Estradiol-3-glucuronide	446.6 -> 271.1	59	183	34	30	67	15	0	0
Estradiol-3-sulphate	350.5 -> 271.1	91861	6549	6128	916	1085	666	130	0
Estriol	522.0 -> 171.0	996118	1470795	712369	706707	2040888	2204481	2168459	2181858
Estriol-16-glucuronide	463.2 -> 287.1	0	0	0	0	0	0	0	150
Estriol-3-sulphate	366.5 -> 287.1	289	2666	1176	1115	1591	629	355	49
Estrone	504.0 -> 171.1	99442	96055	138811	65646	195025	110476	97430	109434
Estrone-3-glucuronide	444.6 -> 113.1	1419	65	0	0	29	0	0	364
Estrone-3-sulphate	348.2 -> 269.1	28558	1461	1238	171	213	172	48	0
Progesterone	315.2 -> 97.1	146840	99228	40227	67844	82797	89967	100085	87391
Testosterone	289.2 -> 97.1	210389	206549	88315	96465	172860	158677	237009	212565

Compound Name	Transition	Washed out with 5% organic Response	Washed out with 10% organic Response	Washed out with 15% organic Response	Washed out with 20% organic Response	Washed out with 25% organic Response	Washed out with 30% organic Response	Washed out with 35% organic Response	Washed out with 40% organic Response
16-Epiestriol	522.0 -> 171.0	333	318	124	3118	1982	420	183	377
16-Ketoestradiol	520.4 -> 171.1	381	607	228	3686	1974	484	218	560
16α-Hydroxyestrone	520.4 -> 171.1	206	461	161	3450	2005	355	136	464
17-Epiestriol	522.0 -> 171.0	591	546	279	4394	3130	744	362	532
17α-Estradiol	506.2 -> 171.1	294	324	178	1805	1820	291	287	401
17β-Estradiol	506.2 -> 171.1	611	607	242	3096	2309	470	393	556
2-Hydroxy-3-Methylether	534.4 -> 171.1	132	206	46	873	1101	137	35	98
2-Hydroxyestradiol	755.0 -> 170.9	32	93	70	62	44	30	28	104
2-Hydroxyestrone	753.4 -> 170.1	154	230	55	395	258	96	144	218
2-Methoxyestradiol	536.4 -> 171.1	81	69	36	797	692	112	89	87
2-Methoxyestrone	534.4 -> 171.1	135	165	80	1226	1236	141	94	168
4-Hydroxyestradiol	755.0 -> 170.9	54	61	32	59	42	35	45	58
4-Hydroxyestrone	753.4 -> 170.1	2146	3217	1047	4997	2108	1151	1956	3437
4-Methoxyestradiol	536.4 -> 171.1	162	130	81	1164	1115	141	87	140
4-Methoxyestrone	534.4 -> 171.1	89	103	51	773	772	137	79	79
Androstenedione	287.2 -> 97.1	0	0	0	0	26	0	0	0
Estradiol-17-sulphate	351.2 -> 97.0	32	526	1740	7843	1961	6770	2664	604
Estradiol-3-glucuronide	446.6 -> 271.1	223	83	561	1129	554	687	520	183
Estradiol-3-sulphate	350.5 -> 271.1	14	820	2541	14430	23758	21548	16146	6549
Estriol	522.0 -> 171.0	1099	9547	291	6367	3458	601	727	10192
Estriol-16-glucuronide	463.2 -> 287.1	73	26	67	130	0	75	22	0
Estriol-3-sulphate	366.5 -> 287.1	52002	41550	54159	93178	57734	48777	31259	2666
Estrone	504.0 -> 171.1	17007	13079	106346	162750	946	17211	5515	9878
Estrone-3-glucuronide	444.6 -> 113.1	51	17	204	353	197	235	159	65
Estrone-3-sulphate	348.2 -> 269.1	0	177	789	4518	6781	6146	4433	1461
Progesterone	315.2 -> 97.1	243	18	39	244	313	27	203	47
Testosterone	289.2 -> 97.1	96	10	72	530	359	86	73	34

Supplementary Table 2. Raw response values obtained from LC-MS/MS chromatogram integration for different SPE washing conditions. Transition refers to the MS1-> MS2 m/z used to detect and give response values. These raw data were used for compiling Figure 2 and Figure 3 in the data article.

Compound Name	Transition	Elution after 5% Wash Response	Elution after 40% Wash Response	Elution after 45% Wash Response	Elution after 50% Wash Response	Elution after 55% Wash Response	Washed out with 40% organic Response	Washed out with 45% organic Response	Washed out with 50% organic Response	Washed out with 55% organic Response
16-Epiestriol	522.0 -> 171.0	356836	729863	836755	672915	455847	223	2739	20673	157605
16-Ketoestradiol	520.4 -> 171.1	500720	1034481	1145101	182481	20139	337	3359	264375	352993
16α-Hydroxyestrone	520.4 -> 171.1	513809	1092373	1117607	71163	6553	1066	9068	363454	383125
17-Epiestriol	522.0 -> 171.0	370841	775117	1069906	798339	745114	241	1993	1249	116551
17α-Estradiol	506.2 -> 171.1	464453	961482	1236281	1204239	1100959	258	2711	313	35
17β-Estradiol	506.2 -> 171.1	491910	1026474	1206509	1274808	1246582	601	3927	636	627
2-Hydroxy-3-Methylether	534.4 -> 171.1	142252	425365	463327	471292	477926	91	598	204	241
2-Hydroxyestradiol	755.0 -> 170.9	75660	268800	177100	236900	70700	4327	4439	4654	3675
2-Hydroxyestrone	753.4 -> 170.1	117010	293780	204100	29498	80660	1105	1558	1526	1657
2-Methoxyestradiol	536.4 -> 171.1	100742	345974	386162	420831	418867	126	635	57	839
2-Methoxyestrone	534.4 -> 171.1	84837	411429	375325	414481	433587	1217	487	1183	4748
4-Hydroxyestradiol	755.0 -> 170.9	40000	266700	188500	241500	56400	321	293	319	201
4-Hydroxyestrone	753.4 -> 170.1	58120	430614	306800	367752	88745	7120	8763	7625	7600
4-Methoxyestradiol	536.4 -> 171.1	104076	470957	533698	622656	574419	126	858	73	54
4-Methoxyestrone	534.4 -> 171.1	236595	754308	886372	883183	845413	1185	1108	1490	4261
Androstenedione	287.2 -> 97.1	338100	416000	456900	496900	567500	183	263	281	317
Estradiol-17-sulphate	351.2 -> 97.0	8187	102	21	0	29	5497	6877	6168	6380
Estradiol-3-glucuronide	446.6 -> 271.1	1468	57	26	0	0	1250	1249	1209	1350
Estradiol-3-sulphate	350.5 -> 271.1	11238	437	113	40	69	14734	14908	10698	10653
Estriol	522.0 -> 171.0	585489	1295951	1493024	65512	5538	3296	63509	403600	438776
Estriol-16-glucuronide	463.2 -> 287.1	3130	28	0	0	0	2246	2900	2940	3073
Estriol-3-sulphate	366.5 -> 287.1	14728	2086	850	344	226	13476	14641	14243	15207
Estrone	504.0 -> 171.1	686020	1109920	938190	987020	875930	33201	40285	45678	23751
Estrone-3-glucuronide	444.6 -> 113.1	7589	173	64	32	24	18448	20080	19127	6513
Estrone-3-sulphate	348.2 -> 269.1	6490	167	59	0	20	7225	6754	5986	7129
Progesterone	315.2 -> 97.1	54292	75547	87283	4173	96174	76	38	80	147
Testosterone	289.2 -> 97.1	53372	68377	77094	124493	127801	36	151	169	180

Supplementary Table 3. Raw response values obtained from LC-MS/MS chromatogram integration for different urine volumes used. Transition refers to the MS1-> MS2 m/z used to detect and give response values. These raw data were used for compiling Figure 4 in the data article.

Compound Name	Transition	5ml urine Response	2ml urine Response	1ml urine Response
16-Epiestriol	522.0 -> 171.0	87	475	451
16-Ketoestradiol	520.4 -> 171.1	2707	9937	10826
16α-Hydroxyestrone	520.4 -> 171.1	151	163	50
17-Epiestriol	522.0 -> 171.0	78	163	304
17α-Estradiol	506.2 -> 171.1	4540	3796	1879
17β-Estradiol	506.2 -> 171.1	1783	2327	1351
2-Hydroxy-3-Methylether	534.4 -> 171.1	4873	2674	989
2-Hydroxyestradiol	755.0 -> 170.9	1423	1501	859
2-Hydroxyestrone	753.4 -> 170.1	102238	100586	69914
2-Methoxyestradiol	536.4 -> 171.1	5274	3915	1778
2-Methoxyestrone	534.4 -> 171.1	7238	4452	2219
4-Hydroxyestradiol	755.0 -> 170.9	773	748	426
4-Hydroxyestrone	753.4 -> 170.1	200	284	218
4-Methoxyestradiol	536.4 -> 171.1	5089	3653	1652
4-Methoxyestrone	534.4 -> 171.1	11607	7474	3238
Androstenedione	287.2 -> 97.1	2091099	2546189	1324310
Estradiol-17-sulphate	351.2 -> 97.0	69173	100423	95463
Estradiol-3-glucuronide	446.6 -> 271.1	3183	2469	1968
Estradiol-3-sulphate	350.5 -> 271.1	2622	2307	2537
Estriol	522.0 -> 171.0	970	1157	1473
Estriol-16-glucuronide	463.2 -> 287.1	1526	8225	12356
Estriol-3-sulphate	366.5 -> 287.1	300	484	2636
Estrone	504.0 -> 171.1	24473	19255	9636
Estrone-3-glucuronide	444.6 -> 113.1	24728	20686	18020
Estrone-3-sulphate	348.2 -> 269.1	104	236	526
Progesterone	315.2 -> 97.1	2991634	2497799	2099428
Testosterone	289.2 -> 97.1	605357	621103	308036

Supplementary Table 4. Raw response values obtained from LC-MS/MS chromatogram integration for different SPE load volumes, and different SPE pH conditions used. Transition refers to the MS1-> MS2 m/z used to detect and give response values. These raw data were used for compiling Figure 5 and Figure 8 in the data article.

Compound Name	Transition	6 ml load volume Response	9 ml load volume Response	12 ml load volume Response	No pH adjustments Response	Buffering at a set pH Response	Washing with lower pH buffer Response
16-Epiestriol	522.0 -> 171.0	2667	2803	2482	3048	2873	1548
16-Ketoestradiol	520.4 -> 171.1	469	501	137	122	58	161
16α-Hydroxyestrone	520.4 -> 171.1	118	19	111	140	26	103
17-Epiestriol	522.0 -> 171.0	76	52	42	40	20	64
17α-Estradiol	506.2 -> 171.1	194	96	120	223	199	306
17β-Estradiol	506.2 -> 171.1	295	317	33	16	24	48
2-Hydroxy-3-Methylether	534.4 -> 171.1	473	95	99	611	709	698
2-Hydroxyestradiol	755.0 -> 170.9	678	649	637	58	28	89
2-Hydroxyestrone	753.4 -> 170.1	69	23	48	233	347	279
2-Methoxyestradiol	536.4 -> 171.1	134	194	576	164	70	233
2-Methoxyestrone	534.4 -> 171.1	109	137	123	91	59	88
4-Hydroxyestradiol	755.0 -> 170.9	111	49	75	58	56	66
4-Hydroxyestrone	753.4 -> 170.1	830	447	278	1061	611	376
4-Methoxyestradiol	536.4 -> 171.1	67	58	39	69	38	21
4-Methoxyestrone	534.4 -> 171.1	212	186	533	300	448	272
Androstenedione	287.2 -> 97.1	1781	952	497	2112	744	751
Estradiol-17-sulphate	351.2 -> 97.0	1713	2661	1501	10249	8564	1196
Estradiol-3-glucuronide	446.6 -> 271.1	336	585	1048	200	45	60
Estradiol-3-sulphate	350.5 -> 271.1	6284	5210	5456	1191	690	207
Estriol	522.0 -> 171.0	186	305	35	676	938	207
Estriol-16-glucuronide	463.2 -> 287.1	9005	5767	9158	5706	4853	2268
Estriol-3-sulphate	366.5 -> 287.1	2267	3123	387	7626	11174	4550
Estrone	504.0 -> 171.1	16	0	40	1043	2245	390
Estrone-3-glucuronide	444.6 -> 113.1	433	4819	8193	396	140	41
Estrone-3-sulphate	348.2 -> 269.1	53	30	38	13	9	10
Progesterone	315.2 -> 97.1	1543	809	202	858	496	702
Testosterone	289.2 -> 97.1	213	421	310	386	504	106

Supplementary Table 5. Raw response values obtained from LC-MS/MS chromatogram integration for different SPE washing and elution volumes used. Transition refers to the MS1-> MS2 m/z used to detect and give response values. These raw data were used for compiling Figure 6 and Figure 7 in the data article.

Compound Name	Transition	3 ml wash volume Response	6 ml wash volume Response	3 ml Elution volume Response	6 ml Elution volume Response
16-Epiestriol	522.0 -> 171.0	633526	998422	1155899	947845
16-Ketoestadiol	520.4 -> 171.1	1019518	1231611	1355127	1120686
16α-Hydroxyestrone	520.4 -> 171.1	1030627	1154125	1386051	1158834
17-Epiestriol	522.0 -> 171.0	744853	984919	905029	865588
17α-Estradiol	506.2 -> 171.1	1096176	1384904	1459751	1428916
17β-Estradiol	506.2 -> 171.1	1016160	1357946	1280093	1204071
2-Hydroxyestradiol	753.4 -> 170.1	25853	30031	3821799	1241094
2-Hydroxyestrone	755.0 -> 170.9	4606	4487	16765	3596
2-Hydroxyestrone-3-Methylet	534.4 -> 171.1	332789	498303	520794	418390
2-Methoxyestadiol	536.4 -> 171.1	249239	362327	411092	323830
2-Methoxyestrone	534.4 -> 171.1	194366	298695	387442	365813
4-Hydroxyestradiol	755.0 -> 170.9	965	1978	26769	16881
4-Hydroxyestrone	753.4 -> 170.1	115570	262860	3820552	1240526
4-Methoxyestradiol	536.4 -> 171.1	269927	408242	657028	574611
4-Methoxyestrone	534.4 -> 171.1	598844	805773	949997	801068
Androstenedione	287.2 -> 97.1	10329	11913	257	255
Estradiol-17-sulphate	351.2 -> 97.0	21	27361	2232	5316
Estradiol-3-glucuronide	446.6 -> 271.1	0	996	204	603
Estradiol-3-sulphate	350.5 -> 271.1	0	38065	5732	1997
Estriol	522.0 -> 171.0	1322583	1619480	1804345	1488990
Estriol-16-glucuronide	463.2 -> 287.1	13	213	436	3396
Estriol-3-sulphate	366.5 -> 287.1	89537	73903	1038	8048
Estrone	504.0 -> 171.1	76289	103686	84594	100742
Estrone-3-glucuronide	444.6 -> 113.1	0	341	4027	1661
Estrone-3-sulphate	348.2 -> 269.1	29	11214	2615	947
Progesterone	315.2 -> 97.1	106538	112422	102520	87069
Testosterone	289.2 -> 97.1	102754	122464	192330	180923

Supplementary Table 6. Raw response values obtained from LC-MS/MS chromatogram integration for different urine volumes used. Transition refers to the MS1-> MS2 m/z used to detect and give response values. These raw data were used for compiling Figure 9 in the data article.

Compound Method Name	Transition	1 ml urine Response	3 ml urine Response
16-Epiestriol	522.0 -> 171.0	419675	298695
16-Ketoestradiol	520.4 -> 171.1	945133	362327
16α-Hydroxyestrone	520.4 -> 171.1	953295	1357946
17-Epiestriol	522.0 -> 171.0	1001833	4487
17α-Estradiol	506.2 -> 171.1	1120444	1978
17β-Estradiol	506.2 -> 171.1	1357923	805773
2-Hydroxy-3-Methylether	534.4 -> 171.1	472900	262860
2-Hydroxyestradiol	755.0 -> 170.9	1516	1619480
2-Hydroxyestrone	753.4 -> 170.1	16301	122464
2-Methoxyestradiol	536.4 -> 171.1	400141	30031
2-Methoxyestrone	534.4 -> 171.1	290835	103686
4-Hydroxyestradiol	755.0 -> 170.9	1203	498303
4-Hydroxyestrone	753.4 -> 170.1	153814	112422
4-Methoxyestradiol	536.4 -> 171.1	583143	408242
4-Methoxyestrone	534.4 -> 171.1	745454	11913
Androstenedione	287.2 -> 97.1	2962	1154125
Estradiol-17-sulphate	351.2 -> 97.0	67	5316
Estradiol-3-glucuronide	446.6 -> 271.1	603	0
Estradiol-3-sulphate	350.5 -> 271.1	212	1997
Estriol	522.0 -> 171.0	1507701	1384904
Estriol-16-glucuronide	463.2 -> 287.1	3396	0
Estriol-3-sulphate	366.5 -> 287.1	8048	624
Estrone	504.0 -> 171.1	87127	116
Estrone-3-glucuronide	444.6 -> 113.1	59	1661
Estrone-3-sulphate	348.2 -> 269.1	947	51
Progesterone	315.2 -> 97.1	47622	984919
Testosterone	289.2 -> 97.1	81125	998422
